# Supplementary material for: Polymer Ligands Derived from Jute Fiber for Heavy Metal Removal from Electroplating Wastewater
Source: Polymers (Basel). 2020 Oct 29;12(11):2521. doi: 10.3390/polym12112521 (PMC7692318; doi:10.3390/polym12112521)
Supplement: Supplementary file 1 [file polymers-12-02521-s001.pdf]

Polymer ligands derived from jute fiber for heavy metal removal from electroplating wastewater

Md Lutfor Rahman *, **Choong Jian Fui**, **Tang Xin Ting, Mohd Sani Sarjadi, Sazmal E. Arshad and Baba Musta**

Faculty of Science and Nature Resources, University Sabah Malaysia, 88400, Kota Kinabalu, Malaysia [2]
* Correspondence: lutfor73@gmail.com

Materials and Methods

Chemicals

Jute known as golden fiber (Tossa jute, *Corchorus olitorius*) and also locally called it Pat in Bangladesh (Figure S1-S5). The jute was obtained from the Machpara, Pansgha, Rajbari, Bangladesh. The fibre is cut into small pieces approximately 0.3 cm in length. Malaysia. Sodium hydroxide (Systerm), glacial acetic acid (J.T. Baker), hydrogen peroxide (QRec), ceric ammonium nitrate (Sigma-Aldrich), methyl acrylate (Sigma-Aldrich), acrylonitrile (Sigma-Aldrich), methanol (Merck), sulphuric acid (Systerm), hydroxylamine hydrochloride (Sigma-Aldrich), Sodium acetate (Systerm), acetone, (Systerm), hydrochloric acid (Merck), copper(II) nitrate (Sigma-Aldrich), nickel(II) nitrate (Sigma-Aldrich), cobalt (II) nitrate (Sigma- Aldrich), chromium(III) nitrate (Sigma-Aldrich). Methyl acrylate and acrylonitrile monomer was purified by columns chromatography with activated alumina. Metal salts and other analytical grade reagents were used without purification.

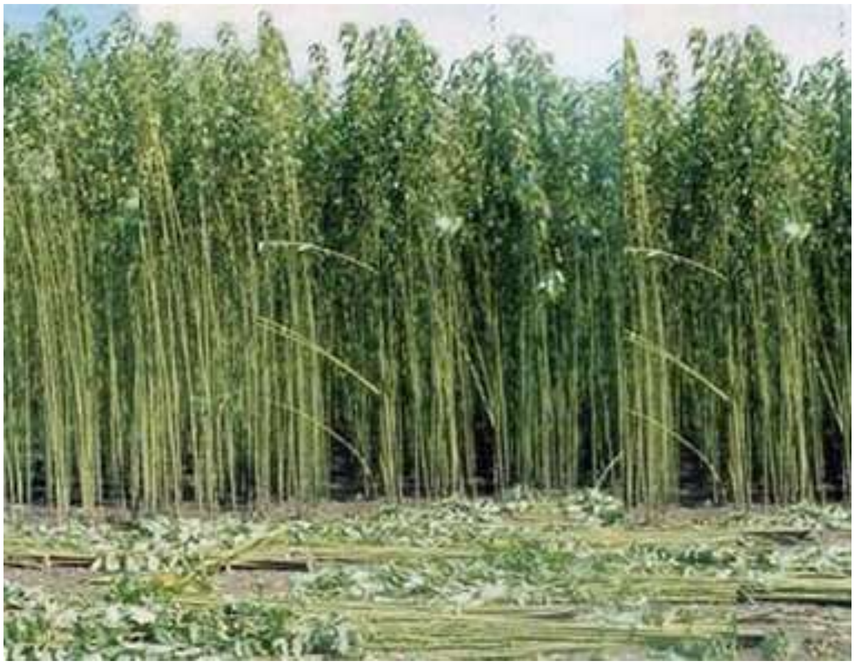

Figure S1. Image shows jute plant and harvesting of jute plant (photo uploaded by Sahidul Hasan Khokon, https://www.indiatoday.in/world/story/bangladesh-gets-international-recognition-for-disclosing-genome-sequence-of-jute-959964-2017-02-10).

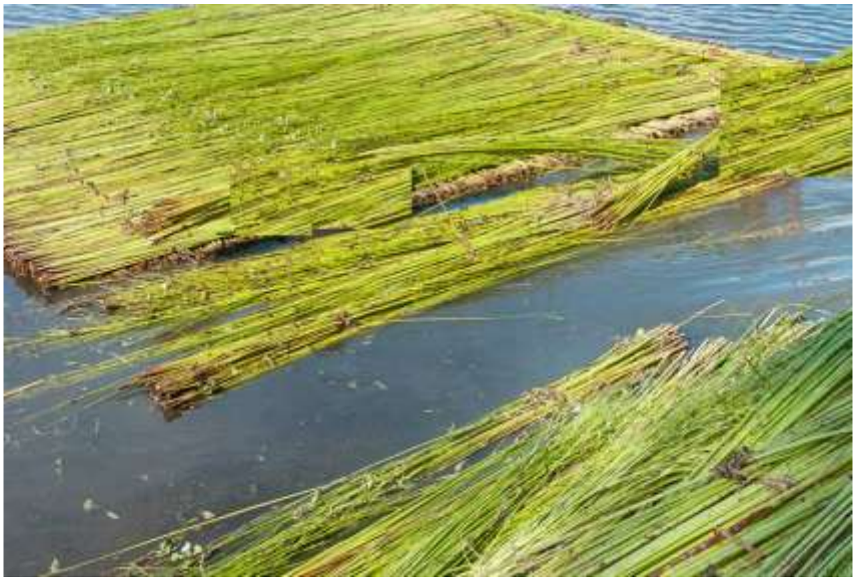

Figure S2. Image shows jute plant dipped into water for rotten (time required 2-3 weeks) (photo uploaded by Sheikh Mofizur Rahman Shipon, http://old.unb.com.bd/special-news/Another-bumper-year-for-jute-on-the-cards-in-Faridpur/46536).

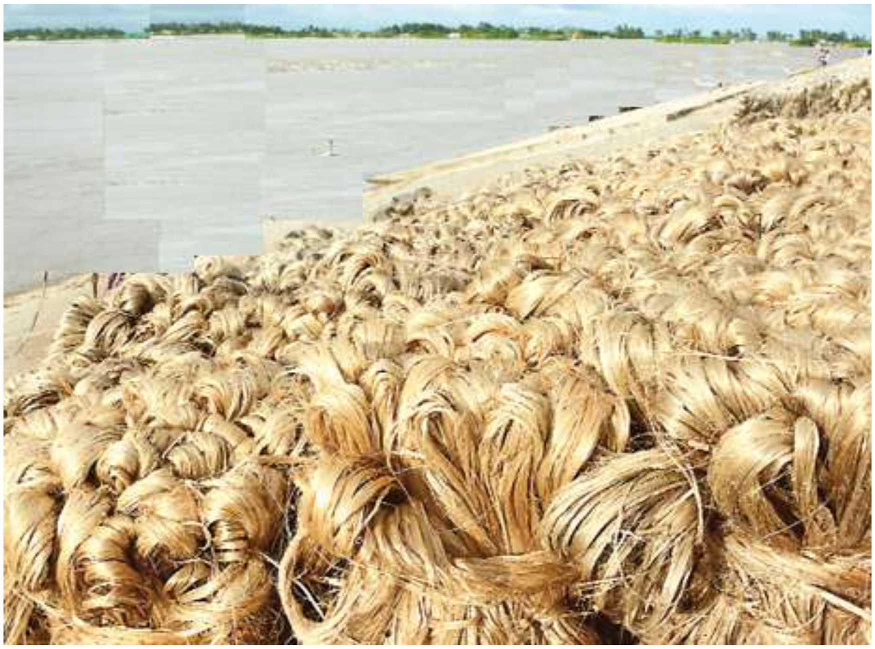

Figure S3. Image shows jute fiber separated from rotten plant and packing for storage (photo uploaded by Monirul Islam Maruf, https://www.daily-sun.com/arcprint/details/242343/A-farmer-is-seen-busy-drying-raw-jute-fibres/2017-07-22)

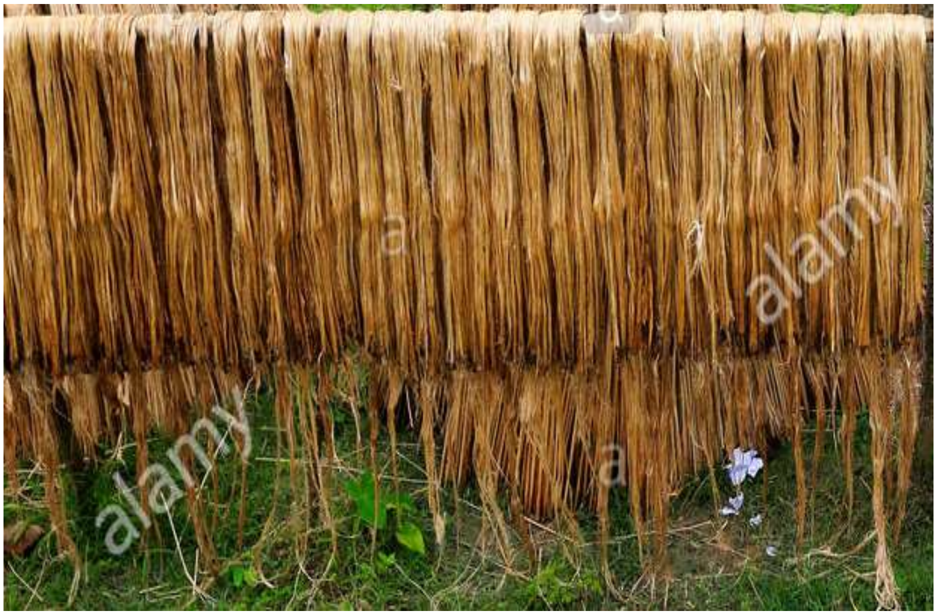

Figure S4. Image shows jute fiber drying process (photo uploaded by Muhammad Mostafigur Rahman, https://www.alamy.com/jute-fibres-and-jute-sticks-are-drying-in-faridpur-bangladesh-image239315383.html)

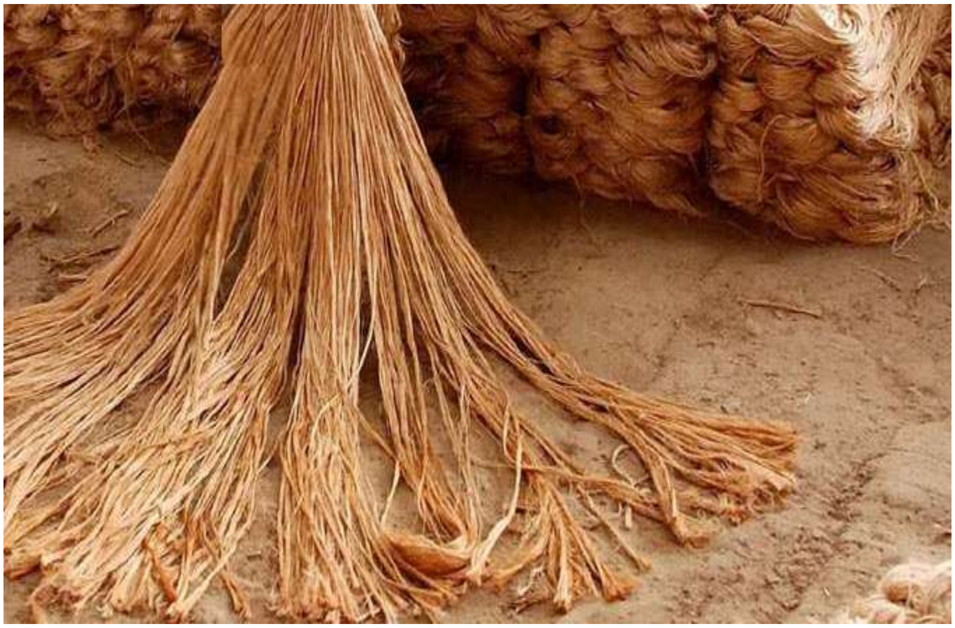

Figure S5. Image shows fully dry jute fiber (photo by FF, https://www.fibre2fashion.com/industry-article/1239/jute-fiber-of-the-future)

Extraction of cellulose from jute fiber

The jute raw fiber was dried in the oven at 60°C. Exactly 250 g of dry jute fiber was boiled with 1 L of 15% sodium hydroxide (NaOH) solution for 2 hours. Then, the cooked jute fiber was then washed with tap water. The washed fiber was boiled again with the 15% sodium hydroxide solution (600 mL) for another 3 h. The crude cellulose washed with water and the soft and brown color of jute cellulose was boiled with 1:1 glacial acetic acid and hydrogen peroxide (250:250 mL) until the cellulose become white color. After the cellulose had turned into white color, the product was then filtered and washed under tap water until both the acid and peroxide were removed. The final product was then being washed again with distilled water followed by drying in an oven for 2 hours at 60°C (Figure S6). The dried cellulose was further used for grafting reaction [1].

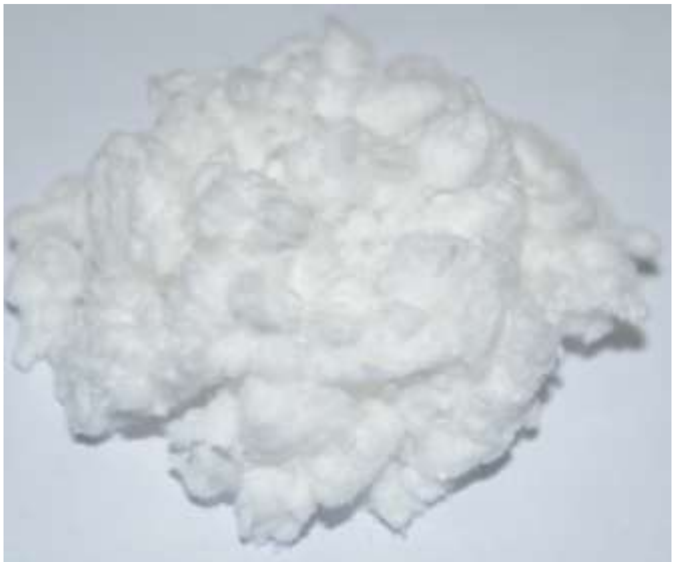

Figure S6. Image shows pure cellulose extracted from jute fiber

Preparation of poly(methyl acrylate) or acrylonitrile grafted jute cellulose

The graft copolymerization was used to synthesize the PMA/PAN grafted cellulose. The purified methyl acrylate (MA) or acrylonitrile monomer was used to direct graft on the cellulose via the free radical process. First, 10.0 g of jute cellulose slurry was prepared with 500 mL of distilled water in 1 L flask. The flask was fixed with condenser, thermometer and magnetic stirrer. The grafting process required absence of oxygen. Therefore, N_2 gas is supplied into the reaction to remove the oxygen. The reaction was conducted with fixed temperature at 60°C. Themixture of cellulose with distilled water was heated at this temperature for an hour. Then, sulfuric acid (2.5 mL of 50%) was added into the reaction for 5 min then solution of ceric ammonium nitrate (2.0 g dissolved into 15 mL water) was added with stirring for 5 min. About 20 mL of methyl acrylate was then added and stirred continuously for 2h. Another batch used 20 mL of acrylonitrile and other reagents and conditions are kept constant. The resultant products were added to the excess amount of methanol (1.5 L, cool to 25°C) into a beaker to precipitate the grafted copolymers. The grafting copolymer was separated and washed the product several times with the alcoholic solution (4:1 v/v methanol and water). The mass of the PMA-grafted cellulose (Figure S7) or PAN-grafted cellulose (Figure S8) was dried in an oven at 60°C to obtain a constant mass [1, 2].

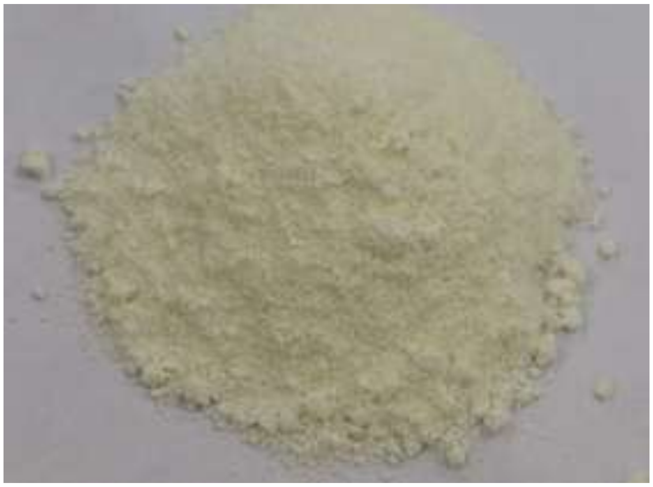

Figure S7. Image shows the PMA-grafted cellulose

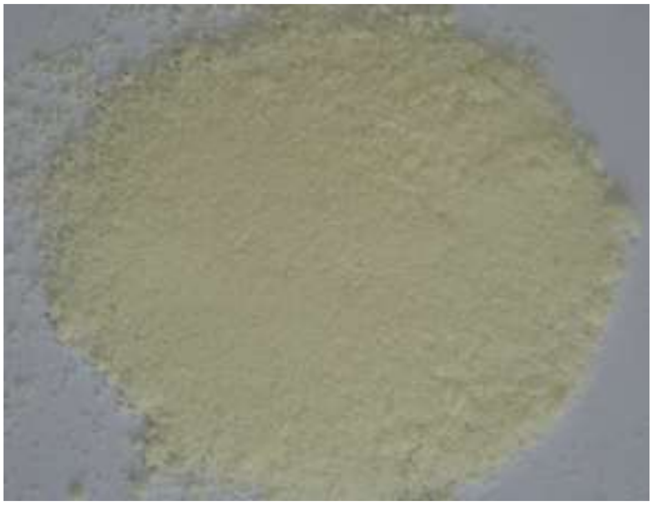

Figure S8. Image shows the PAN-grafted cellulose

Synthesis of poly(hydroxamic acid) ligand or poly(amidoxime) ligand

A hydroxylamine solution was prepared by dissolving 25.0 g of hydroxylammonium chloride ($NH_2OH.HCl$) into 300 mL of a methanol solution (4:1 methanol and water). Then 30% NaOH solution was added drop by drop into the hydroxylamine solution with the formation of NaCl salt until the pH gain to 12. The NaCl salt was filtered off by using filter paper. Then 20.0 g of grafted product (PMA/PAN) was transferred into a reaction flask fitted a condenser, thermometer, and stirrer. The flask was placed on the oil bath with controlled temperature. The hydroxylamine solution was supplied into the reaction and the heterogeneous mixture was agitated by using magnetic stirrer and the temperature was controlled in the range 70-75 °C for 4 h. Once the reaction completed, the cellulose-based poly(hydroxamic acid) (Figure S9) or poly(amidoxime) ligand (Figure S10) was filtered out from the solution followed by washing with methanolic solution (MeOH to H_2O ratio is 1: 4). Both polymer ligands were treated with 150 mL of diluted hydrochloric acid (5%) in methanolic solution for 10 minutes to convert the jute-supported polymeric ligand into hydrogen form polymeric ligands. The ligands were then being filtered out and washed with methanol for several times. Finally, the product was placed and dried in an oven at 50°C to obtain the actual product weight [1, 2].

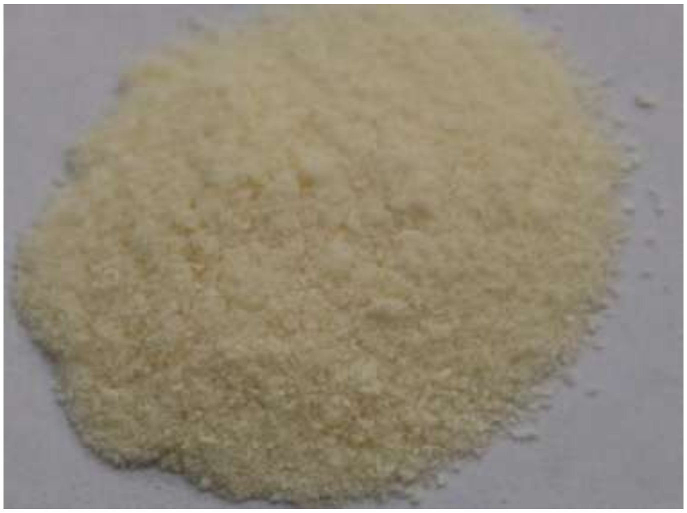

Figure S9. Image shows the poly(hydroxamic acid) ligand

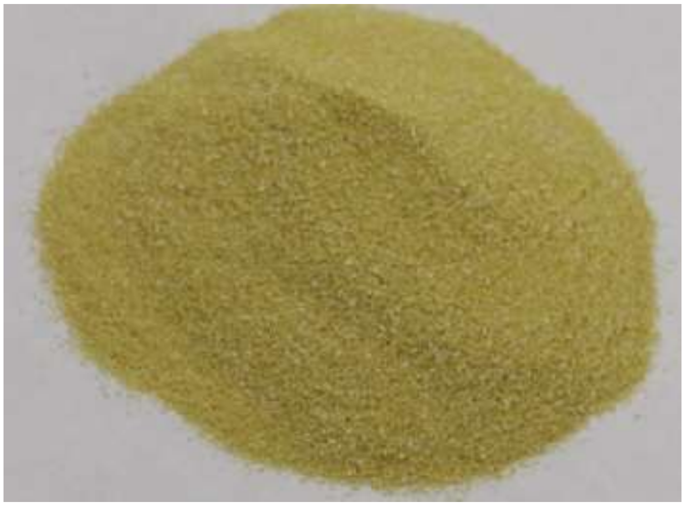

Figure S10. Image shows the poly(amidoxime) ligand

Adsorption of metal ions by PHA/PA ligands

Batch adsorption. The binding behaviour of the synthesized ligands with selected metal ions was studied at different pH from 3 to 6 using batch adsorption technique. The image show PHA complex at Figure S11 and PA complex at Figure S12. For the removal of toxic ions (Copper, Chromium, Cobalt and Nickel), exactly 0.200 g of dried synthesized ligands (PHA/PA) was placed into a series of the centrifuge tube with 15 mL distilled water for 30 min. After that, 5 mL ofbuffer solution (acetic acid was added drop by drop into a 0.1M ammonium acetate until desired pH) ranging from pH 3 to 6 was added to each bottle for 1h. After that, 5 mL of 0.2 M aqueous metal solution was added followed by agitation with controlled temperature for 2h at a continuous agitation rate of 200 rpm. After the adsorption reach to equilibrium, the adsorbent was removed by filtration and the remaining solution concentration of metals ions are determining by ICP-OES. The beginning and final concentration of the metals ions were determined by using the equation 1 [1,3].

$$q_e = \frac{(C_o - C_e)V}{m} \quad (1)$$

where q_e is the adsorption capacity of metal ions (mg g^{-1}). Here C_o and C_e are the initial and the final concentration of the metal ions (ppm) in solution, respectively. V is the volume of the metals ions in solution (L) and m is the quantity of jute cellulose-based adsorbent (g).

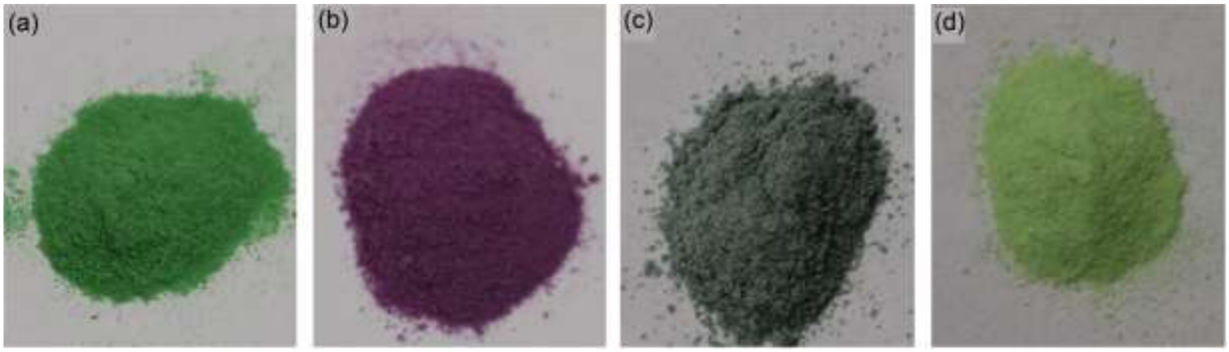

Figure S11. Image shows the poly(hydroxamic acid) complex with Copper (a), Cobalt (b), Chromium (c) and Nickel ions (d).

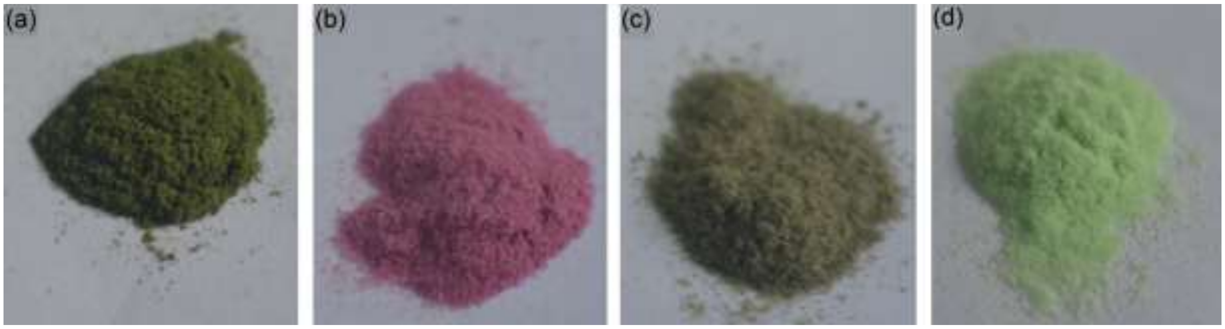

Figure S12. Image shows the poly(amidoxime) complex with Copper (a), Cobalt (b), Chromium (c) and Nickel ions (d).

Sorption isotherm study

For the case of sorption behavior, the conventional batch method was used to conduct the adsorption experiment with constant room temperature and agitation speed at 200 rpm. Exactly 0.200 g of dried poly(hydroxamic acid) ligand (PHA) or poly(amidoxime) ligand (PA) was placed into a series of the centrifuge tube and the ligands was immersing into 15 mL distilled water for 30 min. Exactly 5 mL of buffer solution (acetic acid was added drop by drop into a 0.1M ammonium acetate until desired pH 6) at pH 6 is added to each bottle for 1h. After that, exactly 5 mL of different concentration of metal ions solution (10 to 1800 ppm) was added, and the solution was agitated with controlled temperature for 2 hours under constant 200 rpm agitating rate. Once the reaction reach was equilibrium, the jute cellulose-supported polymer was separated, and then the concentrations of metals ions will then be determined using the ICP-OES. The results can be calculated using the equation 1 [1-3].

Kinetic study

A kinetic adsorption study is carried out using 0.5 g dried ligands (PHA/PA) being immersed in 15 mL distilled water and left for 30 min. Then 5 mL buffer solution pH 6 (ammonium acetate buffer solution) was added into each tube and left for 1h. Then 5.0 mL (0.2M) selected metal ions solution was added and shaken under fixed temperature at different time intervals ranging from 5 min to 120 min under 200 pm agitation speed. After that, the final metal ions concentration was determined by using the ICP-OES. The amount of the metals ions being adsorbed at that particular time was determined using the equation 2 [1].

$$q_t = \frac{(C_o - C_t)V}{m} \qquad (2)$$

where q_t represented the adsorption capacity by ligand at time, t (mg g^{-1}). C_o and C_t are the initial and the final concentration of metals ions (ppm), respectively. L represents the mass of ligand used (g) and V represents the volume of metal ions solution (m).

Characterizations

A Perkin-Elmer FT-IR Spectrometer (Spectrum 100) was used to finding functional groups of intermediate and products. The samples were analyzed using KBr pellets. About 20 mg of sample was mixed with dry KBr pellet and crushed it smoothly and analyzed with FT-IR instrument. The morphological analysis was performed by Field Emission Scanning Electron Microscopy (FE-SEM) with JEOL, JSM-7800F and JSM-7900F. The nanoscale micrograph of transmission electron microscopy (Hitachi instrument (HT-7700) was used to find the metal cluster in the sample. Thermogravimetry analysis was performed for the PMA-grafted cellulose, poly(hydroxamic acid) ligand and poly(hydroxamic acid) ligand with copper-complex with heating rate 10 min^{-1} in N_2 gas (Mettlar Toledo TGA/DSC +3). Inductively Coupled Plasma Optical Emission Spectrometry (Perkin Elmer OPTIMA 7300 DV ICP-OES) was used in this study for determining heavy metal ions concentrations in solution. pH meter (Schwerzenbach 8603) was used to prepare the sodium acetate buffer solution. Oven (Protech ABBI-50) and Shaker machine (Protech model 721) are used in this study. The XPS analyses were performed by using the Scanning X-ray (PHI VersaProbe II) X-ray Photoelectron Spectrometer to find the ionic spices and bonding characters in the samples.

References

1. Rahman, M. L.; Sarkar, S. M.; Yusoff, M. M.; Kulkarni, A. K. D.; Chowdhury, Z. Z.; Ali, M. E., Poly (amidoxime) from polymer-grafted khaya cellulose: an excellent medium for the removal of transition metal cations from aqueous solution. *BioResources* **2016,** *11* (3), 6780-6800.
2. Rahman, M. L.; Fui, C. J.; Sarjadi, M. S.; Arshad, S. E.; Musta, B.; Abdullah, M. H.; Sarkar, S. M.; O'Reilly, E. J., Poly (amidoxime) ligand derived from waste palm fiber for the removal of heavy metals from electroplating wastewater. *Environmental Science and Pollution Research* **2020,** *27* (27), 34541-34556.
3. Rahman, M. L.; Mandal, B. H.; Sarkar, S. M.; Yusoff, M. M.; Arshad, S.; Musta, B., Synthesis of poly (hydroxamic acid) ligand from polymer grafted corn-cob cellulose for transition metals extraction. *Polymers for Advanced Technologies* **2016,** *27* (12), 1625-1636.
